# Spatio-Temporal Features in Action Recognition Using 3D Skeletal Joints

**DOI:** 10.3390/s19020423

**Published:** 2019-01-21

**Authors:** Mihai Trăscău, Mihai Nan, Adina Magda Florea

**Affiliations:** Faculty of Automatic Control and Computers, University Politehnica Bucharest, București 060042, Romania; mihai.trascau@cti.pub.ro (M.T.); mihai.nan@stud.acs.upb.ro (M.N.)

**Keywords:** action recognition, AmI, AAL

## Abstract

Robust action recognition methods lie at the cornerstone of Ambient Assisted Living (AAL) systems employing optical devices. Using 3D skeleton joints extracted from depth images taken with time-of-flight (ToF) cameras has been a popular solution for accomplishing these tasks. Though seemingly scarce in terms of information availability compared to its RGB or depth image counterparts, the skeletal representation has proven to be effective in the task of action recognition. This paper explores different interpretations of both the spatial and the temporal dimensions of a sequence of frames describing an action. We show that rather intuitive approaches, often borrowed from other computer vision tasks, can improve accuracy. We report results based on these modifications and propose an architecture that uses temporal convolutions with results comparable to the state of the art.

## 1. Introduction

Action Recognition is one of the key features for implementing Ambient Intelligent (AmI) systems today. Knowing which action the user is performing at a given time and location offers the means to create systems that react accordingly in a timely fashion.

The classical solution is to use a multitude of sensors embedded throughout the environment or to replace most devices and appliances with ‘smart’ ones, capable of reporting back their interactions with the user. Due to cost, the extensive infrastructure needed, and the technical expertise required to deploy and maintain such systems, the solution quickly becomes intractable.

Instead, Computer Vision techniques have more chances to be able to extract patterns from the behavior of the user, at a fraction of the cost, considering that, for example, it would only require a handful of cameras to cover a typical residence or workplace.

In Ambient Assisted Living (AAL) scenarios, AmI systems are even more sensible to correctly detecting what the user is doing. People with special needs, for example, often require some form of remote monitoring. This is either for measuring and reporting their activity levels or to possibly intervene in case of dangerous situations. For example, fall detection [1] has been a central topic for systems looking to extend the independence of elderly users in their own homes. Moreover, improving personal hygiene, keeping the proper medication schedule, and managing food intake are all part of the Activities of Daily Living (ADL) group of activities which are essential to keep track of activities when dealing with vulnerable users. Action recognition techniques are useful for detecting low-level action patterns that are needed for this type of human behavior analysis problems.

The problem of recognizing human activity using the RGB image extracted from video is not new. It is one of the first problems that computer vision has tried to solve. From the beginning, this problem was considered a challenging one, because there are many variables that need to be taken into consideration: height of the person, the scene in which the action takes place, brightness levels, the angle from which it is viewed, the fact that an action can be executed in a different manner from one person to another, and more. In the past, the information provided by the video cameras has been extensively studied and analyzed as input for systems capable of identifying and recognizing human actions. Initial models designed for action classification used 2D images as inputs on top of which engineered features were extracted and used by classifiers such as Support Vector Machines (SVM—[2,3]) and Hidden Markov Models (HMM—[4,5]). Recently, classifying user actions by means of Convolutional Neural Networks is a major direction of research in the area.

Action recognition based on 3D skeleton joints aims to identify human actions using information from a series of sensors, with the RGB images, depth maps and coordinates of the of the points that make up the human skeleton being the main types of inputs used to perform this operation.

Compared to RGB images, depth maps have the advantage that the regions of interest in the image are easier to segment. Differentiating the silhouette of a person, even if his clothes are very similar with the background, is more robust when using depth data. Moreover, due to optical characteristics of the sensor, depth maps are far less susceptible to brightness variations or low light situations, not least the 3D context obtained through depth maps provides a clear structure of the action scene, which has a positive influence on the accuracy of the learning algorithms due to more information being available at input.

This paper reports work based on this approach: depth-based sensors and data for classifying actions based on 3D motion of skeleton joints. Using the Kinect from Microsoft, an RGB-D sensor capable of providing multimodal data, we investigate how actions may be recognized with high accuracy by using the skeleton joints which the sensor provides, inferred from the depth data. We explore different ways of expressing the input features in combination with the interpretation of the spatio-temporal aspects thus producing different types of architectures for the proposed solutions. In particular, we propose an architecture based on the temporal convolutional network (TCN) presented by Bai et al. [6].

In Section 2 we provide a brief overview of related work, with a focus on various interpretations on what an action means. Section 3 describes different variants of an architecture based on temporal convolutions, with reference to relevant work, as our main contribution. Section 4 presents the results of these proposed solutions. In Section 5 we discuss the major takeaways regarding action recognition based on 3D skeleton joints.

## 2. Related Work

In this section, we focus on the main approaches used to represent the information that describes the human skeleton, specifying for each approach how it can be used to encode a spatio-temporal sequence of skeletal joint points based on which the human action is recognized. The concept of basic human skeleton representation was first introduced by Johansson in 1973 [7], demonstrating that a small number of points can effectively represent the behavior and characteristics of the human body.

One popular RGB+D sensor, capable of providing RGB images, depth maps and infrared (IR) images, is Microsoft’s Kinect sensor. Apart from images, it also provides algorithms to estimate positions of the human skeleton joints, both in 2D (relative to the RGB image) and in 3D. Even though Microsoft recently announced that Kinect will be discontinued, the capabilities of the sensor are now also available for other sensors on the market. Moreover, there are other related computer vision tasks which are also interested in collecting and processing depth information, for example, robotic navigation and mapping. For these reasons, action recognition based on 3D joints is still a current subject of research.

3D skeleton-based representations deliver promising performance, especially for applications running in real time, including games that use Kinect. This representation of human body, based on the coordinates of the points that make up skeleton, can model relationships between joints and codify the whole-body configuration using a very small amount of information.

**Figure 1:**
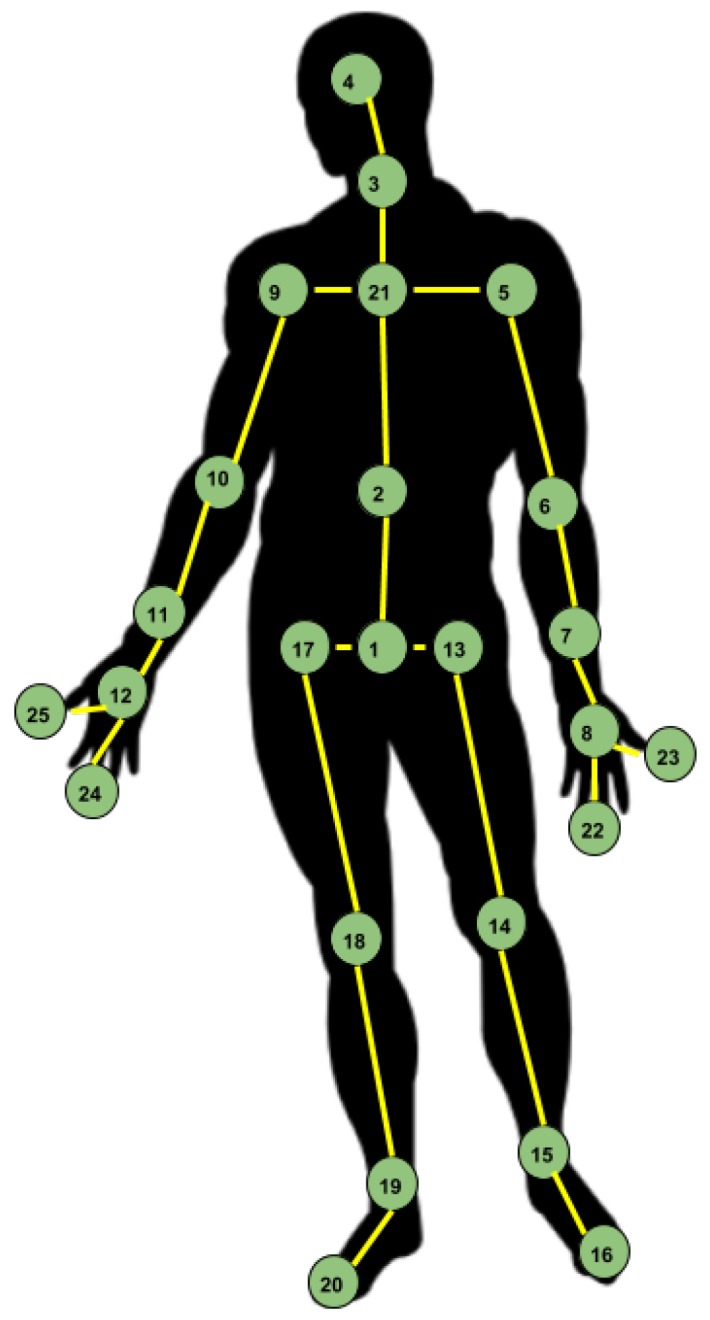
Configuration of 25 body joints proposed in NTU RGB+D Dataset [8]. 1—base of the spine, 2—middle of the spine; 3—neck, 4—head, 5—left shoulder, 6—left elbow, 7—left wrist, 8—left hand, 9—right shoulder, 10—right elbow, 11—right wrist, 12—right hand, 13—left hip, 14—left knee, 15—left ankle, 16—left foot, 17—right hip, 18—right knee, 19—right ankle, 20—right foot, 21—spine, 22—tip of the left hand, 23—left thumb, 24—tip of the right hand, 25—right thumb.

The configuration considered in the presented approaches is the one proposed by the NTU RGB+D Dataset authors [8]. Thus, the human skeleton is composed of 25 points, as presented in Figure 1.

### 2.1. Using Raw 3D Joints Positions

One first approach was to use the coordinates of the joints that make up the human skeleton as input features for a deep neural network classifier. For each action there is a T×N×S tensor, where *T* is the number of frames, *N* is the number of joints and *S* the number of coordinates for each joint [8].

Veeriah et al. [9] proposed to add a new gating mechanism for Long Short-Term Memory (LSTM) to model the derivatives of the memory states and explore the salient action patterns. All the input features were concatenated at each frame and were fed to the differential LSTM at each step.

Zhu et al. [10] introduced a regularization term to the objective function of the LSTM network to push the entire framework towards learning co-occurrence relations among the joints. To ensure effective learning of the deep model, an in-depth dropout algorithm for the LSTM layers was designed, which performs dropout for the internal gates, cell, and output response of the LSTM cell.

The order in which the joints are arranged in the input is important, from the spatial perspective, since just ordering them by index means that there are cases when there is no direct correspondence between two adjacent joints. From this point of view, several approaches have been tried, for example seeing the human skeleton as a tree, rooted in the central joint (the joint with index 1 in Figure 1). By performing a traversal of this tree, the order in which the joints are arranged into the feature vector ensures that any two consecutive joints are adjacent.

The 3D skeleton joints representing the human body can be grouped into five parts: two arms, two legs and one trunk; and human actions can be interpreted as a series of interactions of different parts of the body. The points that form each part of the body move most often together, and the combination of 3D trajectories forms more complex motion patterns.

Starting from this perspective, Hierarchical Models have exploited the inherent structure of the human body and asserted that simple human actions are executed by skeleton components. Using Recurrent Neural Networks (RNNs) for accessing contextual information, Du et al. [11] proposed a hierarchical bidirectional model for the classification of human actions. This model is composed of 9 layers, where each layer is concerned with different structures, having different roles in the proposed architecture. The first layer contains five bidirectionally recurrently connected sub-nets, one for each part of the skeleton, while the second is the fusion layer, which ensures the combination of the five parts. Furthermore, the component parts are fused, from one layer to another, and the result is a representation covering the entire body. The first part of this proposed architecture, stacked Bidirectional Recurrent Neural Networks (BRNNs), can be considered to extract the spatio-temporal features of the skeleton sequences, while the second one, composed of a fully connected (FC) layer and SoftMax layer, performs the classification.

Attention-Based Models have been employed for human action recognition with the idea that identifying key frames and key joints in the sequence will ensure that classification is based mainly on relevant information. Sijie Song et al. [12] have used a model based on spatial and temporal attention, implemented using an end-to-end multi-layered LSTM network. This network can choose the dominant joints for each frame, from the perspective of spatial attention, and assign to each frame a different degree of importance. Considering a skeleton composed of *K* joints, each joint having 3 coordinates, each frame is represented as: $x_{t}={\left(x_{t,1},\ldots ,x_{t,K}\right)}^{T}$; the scores describing the importance of each joint are calculated using Equation (1), where $U_{s},W_{xs},W_{hs}$ are learnable parameter matrices, $b_{s},b_{us}$ are the bias vectors and $h_{t-1}^{s}$ is the hidden variable from an LSTM layer. By passing these scores through a SoftMax layer, the probability for the importance of each joint is obtained. Temporal attention is also important because prediction is more accurate when considering only the frames that compose the essential moment of the action.

(1) $$s_{t}=U_{s}\tanh(W_{xs}x_{t}+W_{hs}h_{t-1}^{s}+b_{s})+b_{us}$$

### 2.2. 2D Spatio-Temporal Interpretations

Convolutional Neural Networks (CNNs) are very good at extracting relevant features for image classification. Their success has inspired researchers to propose bi-dimensional representations of human actions. Researchers have tried to collapse both the spatial configuration of every body pose at each frame, and their individual movements into 2D planes, which are then easy to feed into CNNs for classification.

Liu et al. [13] proposed an approach in which they transformed the joint sequence into a spatio-temporal representation that can be used as input for a CNN. Starting from a coordinate sequence for the skeleton joints, they propose a matrix $I\in R^{H\times W\times 3}$, using a tensor $\psi \in R^{h\times w\times 3}$, obtained by arranging the indexes of the joints in the form of a 2D-grid. For each frame, they have generated *m* matrices in $R^{h\times w}$ with unique elements in range [1,h×w], and used these to generate 2D images. They also enriched their data by adding values for velocity. The features extracted by the neural network were then used to train an SVM classifier.

Yang et al. [14] have proposed transforming the vectors representing the 3D coordinates of joints in the sequence into a 2D image. All values were scaled to the [0,255] range, and the resulting image was re-sized to use a constant size representation for all actions. They considered a fixed order of the joints, determined by a traversal of the tree with root in joint 2 (see Figure 1), in which some joints appear several times. According to this order, the joints were arranged in a line, each line containing 49 joints. Considering that the skeleton image is already capturing the information from the spatio-temporal point of view, they used a two-branch attention architecture, which includes ‘mask branches’ for learning a 2D attention mask, and ‘residual branches’ for refining and reusing features from the previous layers; thus, the network learns attention masks from a single skeleton image. These two branches are merged to output a feature block. Considering that the previous approach had a major limitation, the fact that the height of the image was capped to 224, information was lost for longer actions. To solve this issue, they generated several images by splitting the skeleton sequences into overlapping sub-sequences. They then extracted CNN features from each image and then fed the results through LSTM layers for performing sequence classification.

### 2.3. Graph-Based Representations

Since the representation of a human skeleton is in essence composed of points that are connected to each other, it is natural to perceive it as a graph. Thus, the joints that make up the human skeleton are seen as vertices of the graph and the links between the points become the edges of the graph. At the same time, there is an increased interest in applying neural networks in problems formulated on data represented as graphs, with several proposals already presented in the past few years [15,16,17]. Some of the results for the tasks that have been tackled have surpassed older techniques (not based on neural networks) and proved the potential of these new approaches. Defferrard et al. [18], have proposed a method, based on the spectral theory of graphs, for generalizing convolutions from low-dimensional regular grids (e.g., 2D images) to high-dimensional irregular domains that can be represented by graphs. Kipf et al. [15] have proposed a simplified version of graph convolutional networks (GCNs) which performed better in terms of inference accuracy and training efficiency by simply operating directly on the graphs.

The advantage of using a graph approach for the action recognition task is that the structure chosen for the graph is fixed, the adjacency matrix is constant and only the cost of the edges varies based on the spatial placement of the joints. Thus, from a spatial point of view, this representation is advantageous. Given that to identify an action, we need a sequence of positions of the human skeleton, the information must be arranged in accordance with the structure of the graph. Therefore, for each vertex of the graph, the coordinates of the associated joint must be specified for each frame. Unfortunately, in this approach, the advantages of temporal representation are lost.

Consequently, Sijie Yan et al. [19] have proposed a model that also preserves temporal connections, considering the graph composed of a series of sub-graphs. They assumed that for every graph $G_{i}$ that models the human skeleton for the *i*th frame, a connection exists between each node $v_{i,j}$ and the similar node from the sub-graph of the next frame $v_{i+1,j}$. With this approach, they have managed to obtain state-of-the-art results on the NTU RGB+D dataset. Moreover, their architecture combines graph convolutions with temporal convolutions. They also present a baseline based on TCNs, which is similar but less accurate.

Zhang et al. [20] expanded the approach in which the convolution was applied from the perspective of the nodes that contained useful information, and the edges were used only to specify the relationships between nodes, proposing thus a method called graph edge convolution. To implement this approach, they used a metric to determine the distance for shortest path between two edges: the shortest path between two edges is the one with the smallest number of nodes, and this number is defined as the length of the path. To apply the convolution operation, they have defined the notion of neighborhood for a given edge, $e_{p}q$, as the set of edges with the shortest path to $e_{p}q$ less than *R*. In their experiments, they considered the value of *R* equal to 1. To perform the normalization, in the case of edges with different weights, they used as a normalizing term the number of neighboring edges with the same labeling values.

## 3. Proposed Methods

**Figure 2:**
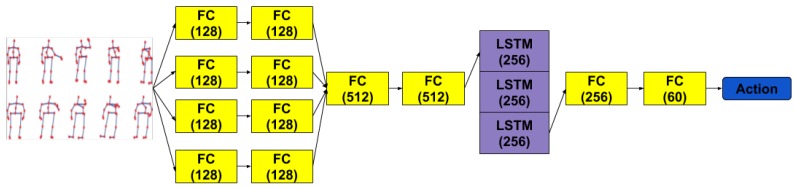
FCs + LSTM - Illustration of the network structure of our first model with FC layers and Stacked LSTM with depth 3. For each layer, the output size is specified.

The first idea we explored to recognize actions from 3D skeletal joints was to directly use the raw frame by frame positions of the 3D skeleton joints. We used a handy treatment of the temporal aspect by first processing the features of each frame and then feeding a sequence of these processed frames to an RNN. Hence, the spatial and temporal dimensions are clearly separated. For the spatial treatment of each skeleton we avoided directly using the raw values and resorted to using several FC layers which run in parallel and recombine later, before reaching the recurrent stage of the network. The reason for using the FC layers is to allow for the network to detect and learn any key combination of joints that might help later in the temporal stage. We connected multiple such layers in the spatial transformation stage to allow for concepts akin to distances between joints (lines), angles between lines, angles between lines and planes and so on, to be potentially discovered by the network. Our attempt was to benefit from the approach of Zhang et al. [21] that do an extensive exploratory search into which types and combinations of features derived from joints are more informative. The intent was to let the network discover them itself, since that would be potentially more robust when learning a completely different set of actions than the one resulted from a hand-engineered process. In treating the temporal dimension, we used LSTM layers to aid in classifying the action sequence. We did experiment with Gated Recurrent Unit (GRU) layers also but found that the LSTMs yielded marginally better results. In this architecture, we increased the depth of the LSTM to 3 levels for the added capacity. Moreover, we used the last third of the outputs of the last LSTM layer as inputs for another stage of FC layers. This draws from empirical observations on our training data, where we have seen that most of the relevant parts of an action happen somewhere in the last third of the sequence. The way recording sessions are scripted for dataset gathering processes means that actors are usually motionless for the first part of the sequence and sometimes just before the sequence ends. The outputs of the last layer are used for computing the cross-entropy loss. The above described architecture is presented in Figure 2.

**Figure 3:**
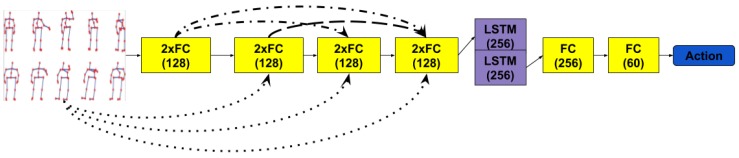
Densely Connected FCs + LSTM—Network structure of the Densely Connected model. The first 4 layers are applied for concatenated input with the output of previous ones.

Continuing the idea of just performing simple transformations on the raw 3D skeleton positions, we have drawn inspiration from the Densely Connected Convolutional Networks proposed by Huang et al. [22]. In this case, the transformations that each layer applies are concatenated with all the inputs of the previous layers. This may allow the recurrent layer to access the original information that the skeleton provided. The temporal stage of the network is treated by using a LSTM layer of depth 2, whose outputs are treated as in the previous architecture before computing the cross-entropy loss. Figure 3 describes this architecture.

Apart from actually computing relations between different joints, we considered that the network would benefit from being provided with movement information localized within a small window around the frame in question, considering the Moving Pose Descriptor proposed in Zanfir et al. [23]. For each joint, $P(t_{0})$, we compute the two derivatives (velocity and acceleration), using the approximations indicated in Equations (2) and (3). These are then concatenated with the coordinates of the joints and passed as input to the neural network. One important step in preprocessing the input data is normalization. In our case, this is performed by subtracting one of the joints of the skeleton from all the others thus placing the origin of the 3D coordinate system in that joint. We used the joint placed at the hip center due to it being the closest to both the geometric center and the mass center of the human body. To increase robustness to varying body shapes of the persons performing the actions, it was necessary to resize each segment that makes up the skeleton of the human body. To perform this operation, the average distance for each segment was determined using the training dataset. Thus, the coordinates of the joint corresponding to the center of gravity were kept constant and the coordinates of the other points were changed, to be able to normalize the distances for each segment. In this way, every body pose was reported to the characteristics of a fictitious individual, chosen as a standard. For parts of our proposed architectures we have performed tests where the inputs are also enriched with the Moving Pose Descriptor.

(2) $$\delta P\left(t_{0}\right)\approx P\left(t_{1}\right)-P\left(t_{-1}\right)$$

(3) $$\delta^{2}P\left(t_{0}\right)\approx P\left(t_{2}\right)+P\left(t_{-2}\right)-2P\left(t_{0}\right)$$

Using the raw features, in our case the x,y,z positions of the 3D joints, grouped in vectors of num_joints×3 components, entails that the latent space obtained after the transformation stage contains all the ‘combinations’ of the joints. This lack of structure at the input loses information on the account that it does not exploit facts like arm or leg joints moving together or having the arm and the leg on the opposite sides of the body moving in opposite directions while walking. In some sense, such a structure was exploited by Du et al. [11] since they processed each major body part separately. However, this prevents somewhat dilutes joints influencing one another across limb, e.g., left hand–right foot. Liu et al. [24] also have an approach where they use the LSTM layers in a 2D layout, where one direction is time and the other is the body joints fed in a sequence. They arranged the body joints according to a tree traversal from a root node (central spine node). This does increase the amount of context information coming into each node but still does not allow composing information from distant nodes.

**Figure 4:**
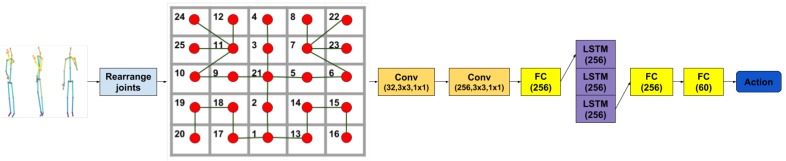
2D Arrangement CNN + LSTM—Illustration of the network architecture based on convolutions on the 2D joint matrix arrangement we proposed. For each convolutional layer, the number of channels produced by the convolution, the size of the convolving kernel and the padding are specified.

We propose a way to add structure to the input features, which comes from our desire to fulfill two goals. The first one is to take into account spatial connections between body joints and the second is to take advantage of the capability of convolutional layers to discover patterns in localized regions while managing to abstract over them in subsequent layers. Therefore, we have proposed an arrangement of the 25 3D body joints into a 5×5 matrix whose corner regions correspond to body limbs while the center holds information on the head and torso. Then, several 2D convolutional layers are applied on the input, completing the spatial transformation stage. We found that a 3×3 kernel size is most appropriate given our input matrix. In addition, we have also tested architectures where we concatenated the initial raw joints to the convoluted features and passed the resulting vector to the temporal stage. The temporal stage of the network is presented in Figure 4.

As we have discussed in Section 2, the graph-like interpretation of the human skeleton joints seems to be the most natural. We have first explored this view by using an implicit representation of graphs, in which we actually study relationships between nodes of the graph. This is similar to the idea presented by the Interaction Networks of Battaglia et al. [25]. In their work they also consider a graph-like structure of the environment, extracting pairwise interactions that would correspond to the edges of this graph (in their case a complete one), with effects of the interactions being approximated via a relational model. Our spatial transformation process follows the same architecture, in the end combining via FC layers the results of each interaction pair into a single feature vector. This vector is then passed to a temporal stage network similar to those already proposed above.

**Figure 5:**
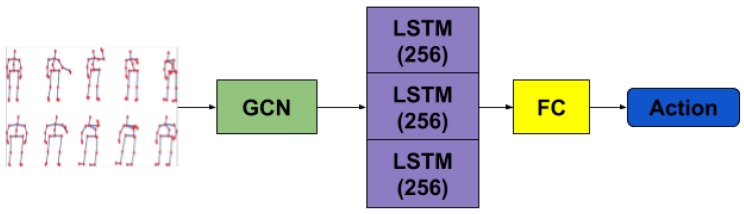
GCNs + LSTM—Network structure of the GCN model with one GCN layer, one Stacked LSTM with depth 3 and one FC layer.

Alternatively, starting from the explicit representation of graphs and the application of convolutions on top of them, inspired by Kipf et al. [15], we replaced the spatial transformation part with GCNs. Adding a layer of graph convolution has allowed for a reduced number of parameters compared to the implicit representation approach, due to not needing to specify a complete graph anymore. Therefore, the spatial representation has access to the degree of bilateral influence the left palm and the right foot have regarding each other. Unlike the approach in [19], we did not take advantage of the hyper-graph-like structure which collapses all body poses in a sequence into a large graph. Instead, we processed the temporal aspect in the same manner as described previously, to measure in a consistent way any advantage the influence of the graph representation of the input may have over the ones used in the architectures presented above. Figure 5 depicts the architecture based on our graph convolutional network.

**Figure 6:**
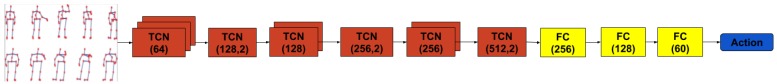
TCNs—Network architecture of the TCN model. For each TCN layer, the number of channels produced by the convolution and the stride (if it is different than 1) are specified. For this model, we used 1D convolutional layers with kernel_size=9 and stride=2 for units with different size for output and stride=1 for others.

In the case of all other proposed architectures we have extensively explored and discussed the spatial stage of our networks. For our main proposed architecture, we reconsidered the temporal interpretation of the actions, especially given findings such as those reported by Bai et al. [6]. The empirical evaluation in their work has pointed out that there are many tasks in supervised learning for which the paradigm of temporal convolutions surpasses that of recurrent networks modeled by LSTMs or GRUs. Therefore, in testing this hypothesis for our case, we replaced the temporal stage of our networks with temporal convolution layers. The output of the last temporal convolution layer is then passed to FC layer reducing it to a size allowing to perform cross-entropy optimization. The architecture is presented in Figure 6.

The performances obtained for the first proposed architecture, namely the one that uses FC layers for feature extraction and LSTM for sequence analysis, indicate that combining even simple spatial transformations and then sequence analysis, using a recurrent neural network, performs strongly. The analysis of the second proposed model, which is a dense one, shows that a too large number of parameters used for spatial transformation actually harms performance. Therefore, we need a better strategy for treating skeleton frames before feeding them to RNNs. We have tried to reduce these inconveniences in our third proposed architecture, which uses joint rearrangement. Because CNNs are analyzing areas of interest with reduced size, the 2D joint arrangement aims to facilitate the detection of relationships between different skeleton joint groups (for example, the left hand may be located far away from the right hand). The idea used in this approach is strong, but the additional information provided by the CNNs is lost when switching to the treatment of the temporal aspect, in our case when feeding the information through LSTMs. In conclusion, this might indicate an essential drawback when decoupling the spatial and temporal dimensions. This can be also deduced from the weak results obtained by Relational + LSTM and GCNs + LSTM, as described in Section 4. Moreover, the implicit graph representation (relational) is not only more computationally costly but yields far weaker results than explicit graphs.

Starting from the aspects observed in the previous approaches, in the TCN-based architecture we did not separate the data from spatio-temporal perspective, but treated them concurrently, using TCN layers. Also, we have not applied major transformations on the input space, leaving the values arranged as a 1D vector. To speed up the training process by reducing the number of epochs needed to learn the optimal parameters for the classifier, we used the Moving Pose Descriptor and we normalized the distance of each segment by their average, calculated for the training dataset. The CNNs, by design, are good for discovering local patterns in the input space. Therefore, before applying the first convolution layer that reduces the number of input channels, we have rearranged the joints. This has been performed using a pre-ordering traversal of the tree with the root set in joint with label 2. This traversal helps strengthening the local interaction between joints.

**Figure 7:**
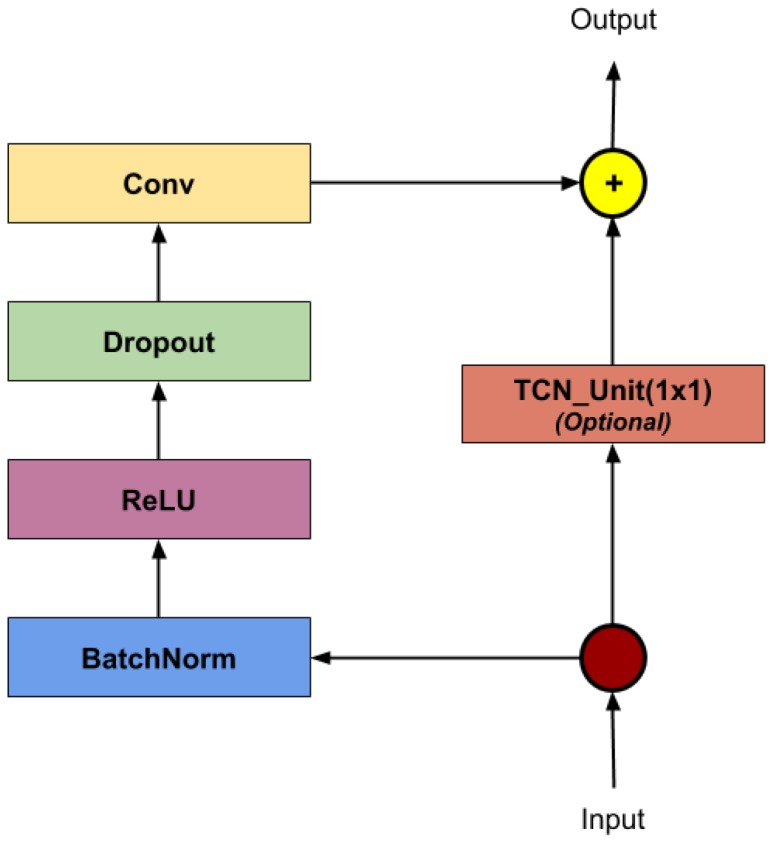
TCN Layer—Architectural elements in a TCN layer. The optional block is used only when the number of channels between the input and the output differs.

To analyze the sequence of joints from a temporal perspective, we used several TCN layers. The architecture of such a unit is shown in Figure 7. To reduce over-fitting, we introduced a dropout layer with probability 0.5 at the latter stages of the model.

We have explored two variants of this architecture. The first is based on one-dimensional inputs, where all 9D joints (3 values for coordinates, 3 values for velocity and 3 values for acceleration) are passed in a single vector, which are then passed to a 1D convolutional layer to reduce the number of input channels. The result of the convolution layer is passed through a series of TCN, average pooling and FC layers with a dropout layer applied at the end. For this model, the TCN layers were implemented using 1D convolutional layers. The second variant arranges the joints in the same layout of a 5×5 matrix which is then fed the 3D convolutional layers. The rest of the model has been preserved, but the implementation of a TCN layer has been changed, replacing the 1D CNNs with 3D CNNs. However, rearranging joints in a 2D grid and using 3D CNNs did not actually help obtaining better results.

Due to our intent to use the human action recognition classifier within a pipeline that must run in real time and with low computational costs, we have also proposed a TCN-based architecture with a reduced number of network parameters without sacrificing much accuracy. This “light” architecture was obtained by removing the last two TCN layers from the architecture shown in the Figure 6.

## 4. Results

### 4.1. NTU RGB+D Benchmark Dataset

To validate the proposed models for action recognition we used the NTU RGB+D dataset [8], which includes over 56,000 sequences, representing a series of 60 possible actions: 50 actions performed by one person and 10 actions performed by two persons. This dataset includes two protocols proposed for testing: cross-subject and cross-view.

In the cross-subject test protocol, one half of the subjects are used for training and the remaining subjects for testing. In the second evaluation protocol, $\frac{2}{3}$ of the viewpoints are used for training and $\frac{1}{3}$ for testing.

**Figure 8:**
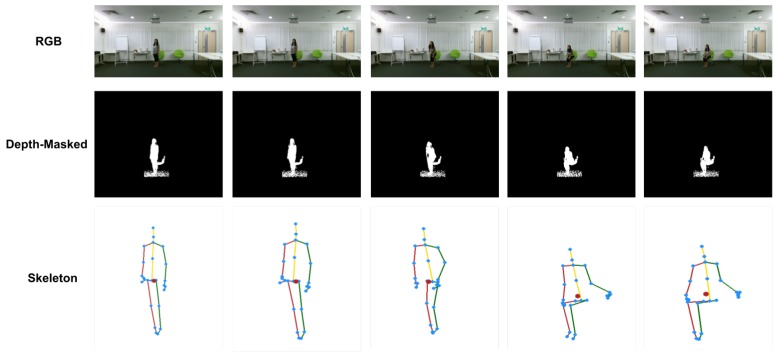
Sample frames of the NTU RGB + D dataset.

The dataset contains 60 action classes in total, which are divided into three major groups: 40 daily actions (drinking, eating, reading, etc.), 9 health-related actions (sneezing, staggering, falling down, etc.) and 11 mutual actions (punching, kicking, hugging, etc.). 40 people were invited to perform the actions collected in this dataset, with ages of the subjects between 10 and 35. To ensure invariance to perspective three cameras were used to record simultaneously during data collection, with two of the cameras being placed at 45° on either side of the central camera [8]. A sample from the dataset is presented in Figure 8.

Analyzing the coordinates of the joints provided by the dataset for a series of actions and using a skeleton viewing tool, we have discovered some inconsistencies. For example, if chairs appear in the scene, these can sometimes be perceived as skeletons. Also, the reflections of some person in windows or TV panels are detected as being separate persons for which skeletons are then computed. For actions involving the interaction of two people, their skeleton indices could be interchanged. To overcome this problem, we determined the skeleton coordinates with index *i* at step *t* by choosing the closest skeleton of the one with index *i* from step t−1.

Because some joints out of the 25 proposed in the dataset are usually quite noisy and not so relevant for the available actions, we tried to reduce the number of joints, ignoring those for fingers and those at the feet. Therefore, we also performed some experiments using only 17 joints out of 25 for the human skeleton.

### 4.2. Experimental Results

Using the data representation approaches and the neural network architectures presented in Section 3, a series of experiments were carried out, and their results are presented in the Table 1.

For the actions involving two people, the model was trained from the perspective of each participant and the result was determined as the arithmetic mean of the action classification scores for each of the two individuals. In an alternate approach, which we abandoned, we determined the most active skeleton of the sequence where two persons were involved and used just this skeleton during training and testing, but the results obtained by this approach actually decreased.

Because the number of frames differs from one action to another, we used the maximum number of frames for a sequence describing an action, 300, and, in the case of shorter actions, we completed the data with zero values. We also considered an alternate interpretation in which each action is described by a sequence of 50 frames. Because from one action to another or from one sample to another the essential moment of the action is placed differently over time, we randomly extracted 50 frames describing the action at each epoch. For this operation, we divided a sample in *p* ranges, where $p=[\frac{num\_frames}{50}]$, and from each range we selected one frame, using uniform selection of a random element. In this way, we can achieve generalization, as an action may take less or more time, depending on the person who executes it. Moreover, taking into account that each sequence was recorded at 30 frames per second and that the average sequence length in the dataset is 82, not much information is lost due to sub-sampling. Therefore, for the most part, we have obtained better results using the smaller rather than the larger sequences.

Considering the work of Zanfir et al. [23], we have tried to determine if the noise which occurred when the data was collected has any adverse effects. The sources of noise are multiple and most of them are related to the Kinect sensor. Thus, to reduce noise, a Gaussian filter was applied over the joints coordinates of each frame, considering a window of 5 frames. Analyzing the results obtained after applying the Gaussian filter, together with the preprocessing inspired from the Moving Pose Descriptor described in Section 3, we noticed no significant increase in the performance of the classifier. However, it did help the model learn much faster, almost halving the number of epochs necessary to reach the best accuracy.

During experiments, we have tried several types of optimization methods and varied parameter values, but the best results were obtained using the Adam optimization method [26], with learning rate values of $10^{-3}$ and $10^{-4}$. As the results show, TCNs have proved to be the best solution of the different architectural variants proposed. It is worthy to note that the simple architecture of FCs + LSTM does not lag behind too much in terms of accuracy, having the added advantage of being a much simpler architecture with a reduced number of parameters. We can observe high drops in accuracy if we use graph convolutions on the input, for each skeleton in the sequence, and then feeding the LSTM (GCNs + LSTM). In contrast to typical graph architectures which obtain state-of-the-art results on this problem, our graph-based model suffers from not encoding the whole sequence into the same graph, a problem which the TCN-based model does not have since every layer has access to the entire sequence. Results for all other architectures are emphasizing the stated intuitions presented in Section 3 regarding the strengths and weaknesses of each approach. The lessons learned from the other proposed architectures have enabled us to achieve very good results based on TCNs.

Our proposed method based on TCNs has been able to achieve comparable results to the state of the art, as described in Table 2. It was able to get better results than [30] which use 2D image representations for the actions. The results in [14] are better than ours, but the complexity of their solution, including the number of parameters, is increased due to the global and sub-sequence attention mechanisms they introduce. The results of their ablation studies indicate that, without the attention sub-networks, the accuracy drops significantly, below our solution. On the other hand, even though our reported results are above those reported for the temporal convolution baselines in [31], expressing the 3D skeleton sequences as a single graph and using graph convolutions does yield better results than those of temporal convolutions. This is likely because each joint is directly connected to its past and previous values, unlike the TCNs. It is to be noted though that in [31] the use of graph convolution layers in the construction of the network is intermittent with temporal convolutions, therefore increasing the number of parameters of the network.

Although accuracy results are slightly below those obtained by methods based on graph convolutions, the less complex architecture we propose has the potential to better suit our purpose, namely finding a model which interprets sequences of actions and composes them into different activities. For example, in the ROBIN-Social project (http://aimas.cs.pub.ro/robin/en/robin-social/) for assistive robots, one goal is to integrate the proposed model into a larger model or a pipeline responsible for detecting the actions of an elderly person from a large number of different actions, and be able to spot when an action actually starts and ends given a continuous stream of images (the robot having the goal to understand what the person is doing and assisting her). In such a case, it is expected that the network will grow considerably to be able to successfully fulfill these tasks, thus guiding our research towards less complex and more supple architectural models. In this regard, the “light” version of our TCN-based method sees a 3.5× reduction in the number of parameters and a 2× reduction for inference speed as compared to [31]. This allows our model to be deployed in more constrained scenarios, such as on assistive robots with limited hardware capabilities, or in setups where multiple cameras are deployed, and action recognition is performed simultaneously on each data stream. For our size and performance comparison we did not take into account networks that use different modalities (e.g., RGB images or depth images) or which do not have any implementation published and available.

## 5. Conclusions

If we analyze the results obtained over the past two years by action recognition methods based on 3D skeleton joints the registered progress is consistent. Larger datasets, which previously seemed to pose multiple problems to all methods, are now slowly becoming insufficient. Most of the methods recently used are based on different CNN and recurrent network architectures, as presented in Section 2. The advances flow from understanding which feature space representations are actually meaningful and informative when using neural networks to recognize human actions. Exploiting the interaction between the spatial and temporal dimensions has yielded better results. This is also the case of our proposed TCN-based architecture, as our experimental results show.

In this paper, we have presented our design decisions and the experimental results based on different convolutional neural network architectures and their variants to recognize human actions using 3D skeletal joints, with the intent to identify a less complex and more supple model, which has the potential to be extended to a larger number of actions. We have also investigated the impact of different input features and associated representations on the models and the accuracy of the obtained results.

Our main contribution is the TCN-based architecture using the extended joint descriptor, for which we obtained results comparable to those of state-of-the-art methods, but using a simpler model, which requires a smaller number of parameters as compared to other performant approaches.

The results in the reviewed literature indicate that graph-based architectures manage to better capture the dynamics of a moving skeleton than some of the other proposed solutions. However, to integrate action recognition in a real-world solution of an assistive robot, as is the case of the ROBIN project for example, the TCN-based model seems more appropriate.

For future work, we have two directions of research. The first one relates to extending our proposed approach to multi-user scenarios, which implies the capability of extracting multiple individual skeletons from the scene and consistently tracking them throughout the actions sequence. One major difference from the actions provided in the NTU dataset is that having multiple persons in the scene does not imply that the performed action involves everyone. Thus, people need to be tracked both separately and together to determine if their action is performed “solo” or in groups.

The second direction of future developments is to combine our proposed architecture with techniques for extracting human pose from RGB and depth data. Moreover, we would like to be able to analyze a given stream of images and signal the frame where an action most likely started or ended, taking into consideration that the length of actions always varies (between different types of actions, persons or even repetitions). This is necessary to achieve our final goal, namely understanding complex human activities composed of several correlated actions, as required by AAL applications.

## Figures and Tables

**Table 1:** Experimental results for the different proposed architectures.

Architecture	Sequence Size	Accuracy	
		CS	CV
FCs + LSTM	300	72.04%	79.41%
FCs + LSTM	50	72.16%	80.39%
FCs + LSTM + Moving Pose Descriptor	50	73.11%	79.06%
Densley Connected FCs + LSTM	300	70.80%	78.34%
Densley Connected FCs + LSTM	50	68.20%	74.07%
Relational + LSTM	50	65.42%	67.46%
Relational + LSTM + Initial data	50	66.14%	69.44%
2D Arrangement CNN + LSTM + Initial data	50	71.48%	77.11%
2D Arrangement CNN + LSTM	50	71.62%	79.18%
2D Arrangement CNN + LSTM + Initial data	300	73.85%	77.31%
2D Arrangement CNN + LSTM	300	74.19%	76.89%
Graph Conv + LSTM	50	63.00%	62.87%
TCNs	50	77.30%	82.47%

**Table 2:** Reported results on the NTU RGB+D Dataset as compared to other methods. Methods in bold font use different modalities of action recognition based on RGB or depth sequences, unlike the rest which only use 3D skeleton joints.

Method	Cross-Subject	Cross-View
Lie Group [27]	50.1%	52.8%
Dynamic Skeletons [28]	60.2%	65.2%
Hierarchical RNN [11]	59.1%	64.0%
Deep RNN [8]	56.3%	64.1%
Part-aware LSTM [8]	62.9%	70.3%
ST-LSTM (Tree) + Trust Gate [29]	69.2%	77.7%
Frames + CNN [30]	75.73%	79.62%
Clips + CNN + Concatenation [30]	77.05%	81.11%
Clips + CNN + Pooling [30]	76.37%	80.46%
Clips + CNN + MTLN [30]	79.57%	84.83%
ST-GCN [31]	81.5%	88.3%
BPLHM [20]	85.4%	91.1%
TSSI + GLAN + SSAN [14]	82.4%	89.1%
**DSSCA-SSLM** [32]	74.86%	-
CNN + Motion + Trans [33]	83.2%	89.3%
**Fusion All** [34]	87.08%	84.22%
FCs + LSTM (ours)	73.11%	79.06%
Densley Connected FCs + LSTM (ours)	70.8%	78.34%
Relational + LSTM (ours)	66.14%	69.44%
2D Arrangement CNN + LSTM (ours)	74.19%	76.89%
GCNs + LSTM (ours)	63.00%	62.87%
TCNs (ours)	77.30%	82.4%
TCNs-light (ours)	77.03%	79.98%
